# The Emerging Regulation of VEGFR-2 in Triple-Negative Breast Cancer

**DOI:** 10.3389/fendo.2015.00159

**Published:** 2015-10-09

**Authors:** Xiaoxia Zhu, Wen Zhou

**Affiliations:** ^1^Molecular Oncology Program, Division of Surgical Oncology, DeWitt Daughtry Family Department of Surgery, University of Miami Miller School of Medicine, Miami, FL, USA; ^2^Department of Biological Science, Columbia University, New York, NY, USA

**Keywords:** VEGF, VEGFR-2, VEGFR inhibitor, JAK2, STAT3, mutant p53, breast cancer

## Abstract

Vascular endothelial growth factor-A (VEGF) signals vascular development and angiogenesis mainly by binding to VEGF receptor family member 2 (VEGFR-2). Adaptor proteins mediate many VEGFR-2’s functions in the development of blood vessels. Cancer cells secrete VEGF to activate VEGFR-2 pathway in their neighboring endothelial cells in the process of cancer-related angiogenesis. Interestingly, activation of VEGFR-2 signaling is found in breast cancer cells, but its role and regulation are not clear. We highlighted research advances of VEGFR-2, with a focus on VEGFR-2’s regulation by mutant p53 in breast cancer. In addition, we reviewed recent Food and Drug Administration-approved tyrosine kinase inhibitor drugs that can inhibit the function of VEGFR-2. Ongoing preclinical and clinical studies might prove that pharmaceutically targeting VEGFR-2 could be an effective therapeutic strategy in treating triple-negative breast cancer.

## Introduction

Vascular endothelial growth factor-A (VEGF-A, also known as vascular permeability factor) is a major factor in regulating functions of endothelial cells in vasculogenesis and angiogenesis (1, 2). VEGF family consists of five members, VEGF-A, VEGF-B, VEGF-C, VEGF-D, and placenta growth factor (3). This review focuses exclusively on VEGF-A since this isoform of VEGF is the most extensively studied, and hereafter, VEGF refers only to VEGF-A. The importance of VEGF in endothelial cells has been demonstrated by mouse models in which both *VEGF-A*^−/−^ and VEGF-A^+/−^ are embryonic lethal, and the mouse embryos died at embryonic day E9.5 and E11, respectively (4, 5). Solid tumors secrete VEGF to induce endothelial cells forming blood vessels in order to gain adequate blood supply for tumors (6). Blood vessel formations further stimulate tumor proliferation and metastasis (7–10). In breast cancer, the expression of VEGF correlates well with decreased overall survival and disease-free survival (8).

**Figure 1:**
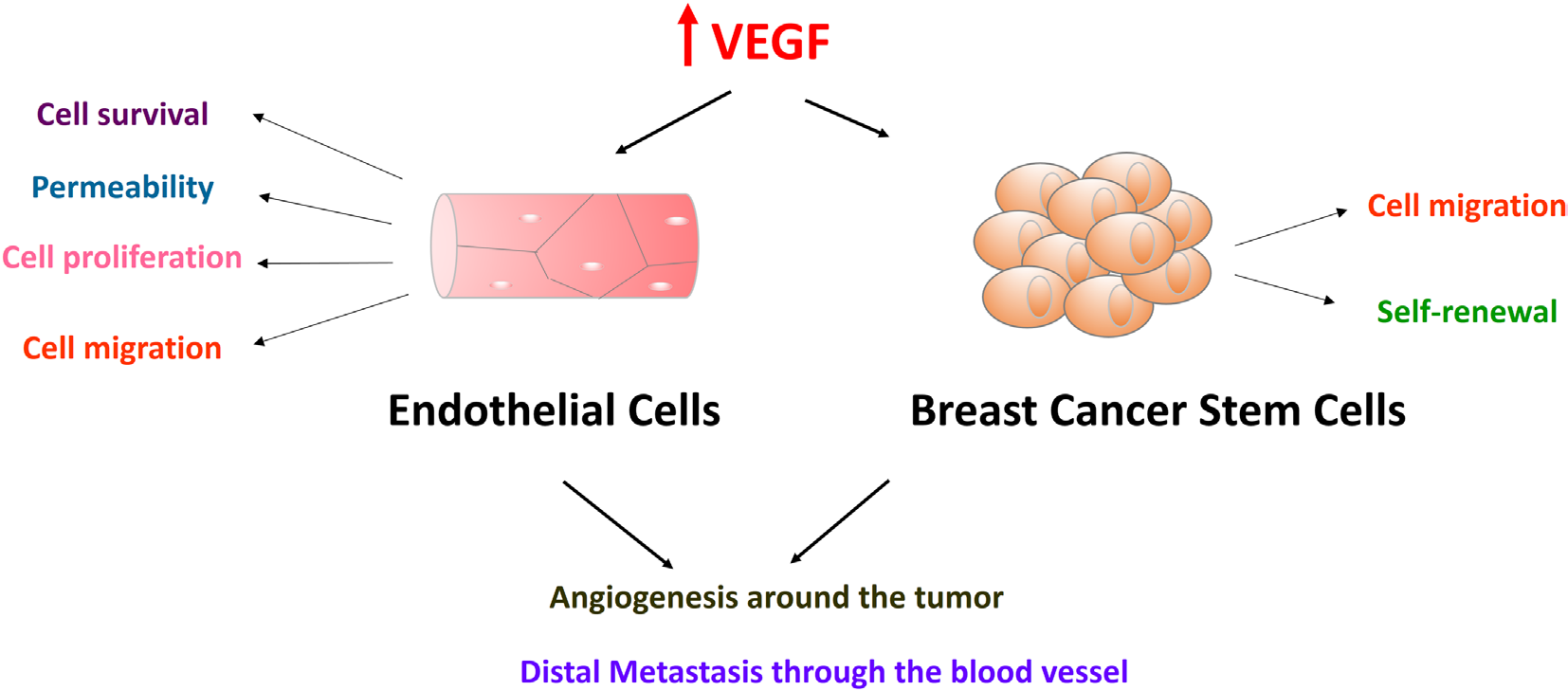
**Vascular endothelial growth factor-A in endothelial cells and breast cancer stem cells promote breast cancer progression and metastasis**. VEGF receptors are expressed in both endothelial cells and breast cancer stem cells. Endothelial cells form endothelium with tight cell–cell junction. Effects of VEGF on endothelial cells include cell survival, permeability, cell proliferation, and cell migration. VEGF also plays an important role in breast cancer cell migration and cancer stem cell self-renewal. These functions converge on promoting breast cancer progression and metastasis. Endothelial cells will be attracted and activated by high levels of VEGF proteins in the tumor-stromal niche. After endothelial cells form new blood vessels surrounding tumor cells, tumor cells will be stimulated by the cytokines in the blood supply. Tumor cells go through epithelial-to-mesenchymal transition, intravasation, circulation, extravasation, and finally form distal metastases.

Current data reveal many facets of VEGF function diversity in both normal and cancer cells, and some of these VEGF functions in promoting breast cancer are depicted in Figure 1. Apart from its well-known role in angiogenesis, VEGF plays a critical role in stem cell maintenance. VEGF has been shown to be important for stem cells in hemopoietic, endothelial, muscle, cardiac, neuronal, and adipose tissues (3, 11–17). Recently, VEGF was found to regulate cancer stem cells (CSCs) self-renewal in brain, lung, and breast tumors (18, 19).

Vascular endothelial growth factor, as a ligand, executes its functions through VEGF receptors. In humans, there are at least three VEGF receptors, VEGFR-1 (20, 21), VEGFR-2 (22), and VEGFR-3 (6, 23). VEGFR-2 is the principal VEGFR in humans (24). It is abundantly expressed in vascular endothelial cells and lymphatic endothelial cells (25). VEGFR-2 is also expressed in neuronal cells, megakaryocytes, hematopoietic stem cells, and different cancer cells (26–30). This review discusses the relevance of VEGFR-2 in breast cancer, particularly in breast cancer CSCs. We further discuss the mechanism through which mutant p53 activates *VEGFR-2* gene expression in breast cancer. The therapeutic implications of these findings for breast cancer are also discussed.

## VEGFR-2 Signaling in Endothelial Cells

**Figure 2:**
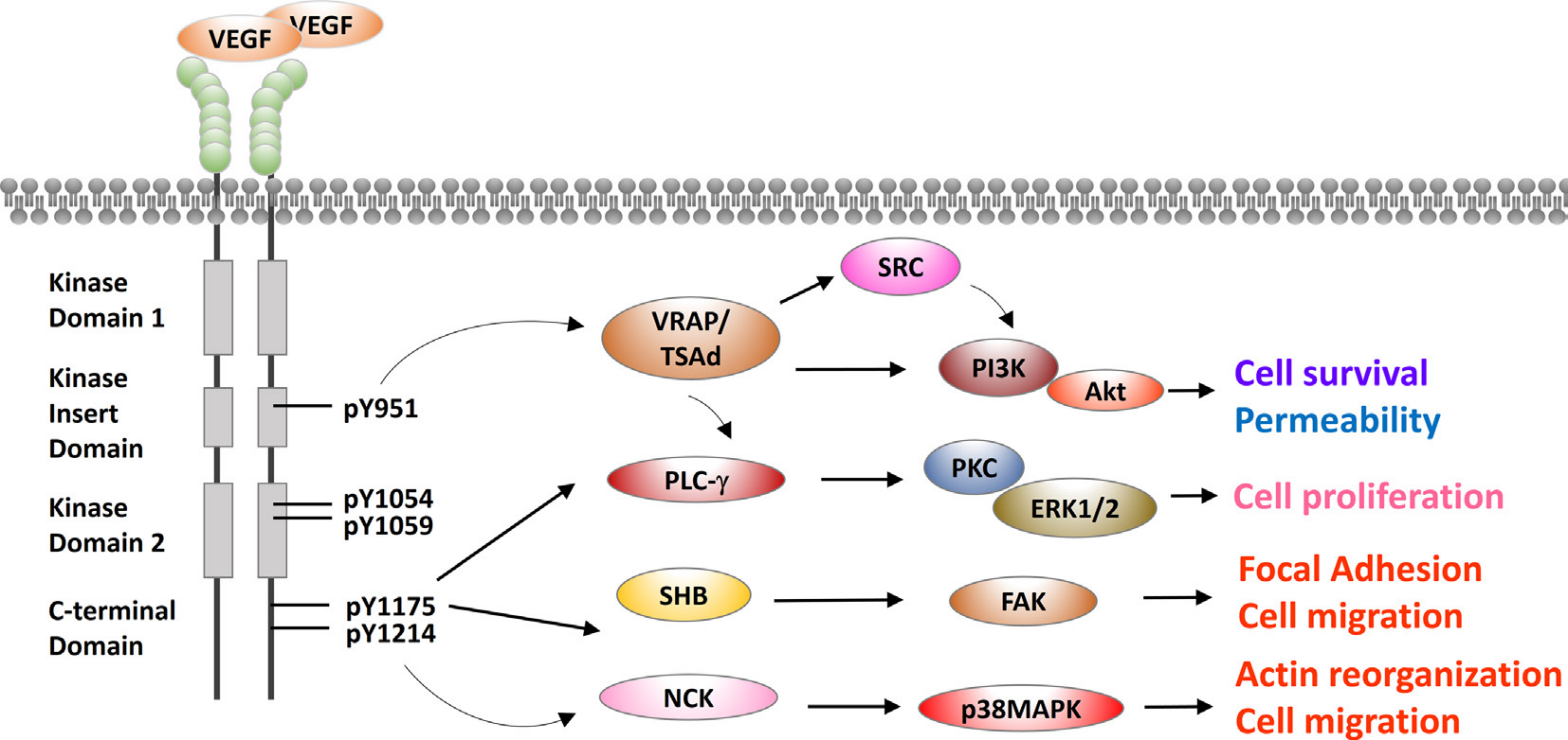
**Cross talks between VEGFR-2 and other signaling pathways in endothelial cells**. VEGFR-2 is presented in a typical receptor tyrosine kinase scheme with an extracellular domain, a juxtaposed transmembrane domain and intracellular kinase domains. Extracellular domain of VEGFR-2 is composed of seven IgG-like domains to bind to its cognate ligand VEGF. Intracellular domain has two tyrosine kinase domains, which are split by a kinase-insert domain of 70 amino acids. Five major phosphorylation residues Y951, Y1054, Y1059, Y1175, and Y1214 are labeled. SH2 domain-containing adaptor proteins are recruited by these phosphorylated tyrosine residues, including VRAP/TSAd, PLC-γ, SHB, and NCK. These adaptors mediate the downstream effects of VEGFR-2, including cell proliferation, permeability, cell survival, and cell migration.

Vascular endothelial growth factor receptor-2 is a receptor tyrosine kinase and a master node in VEGF signaling. VEGFR-2 has an extracellular portion consisting of seven immunoglobulin-like domains, a transmembrane domain, and an intracellular portion containing two tyrosine kinase domains (31). A kinase-insert domain splits its two tyrosine kinase domains. VEGF binds to and triggers two VEGFR-2 monomers to dimerize and to be autophosphorylated (3, 32, 33). A major phosphorylation site Y951 is in the kinase-insert domain (34). Four other major sites are Y1054/Y1059 in the tyrosine kinase domain and Y1175/Y1214 in the C-terminus (35, 36). Properly phosphorylated tyrosine residues serve as a binding surface for SRC homology 2 (SH2)-domain-containing adaptor proteins, as discussed below and depicted in Figure 2.

### VEGF-Receptor-Associated Proteins/T-Cell-Specific Adapter Molecule

In human umbilical vein endothelial cell models, VEGF stimulation triggers VEGFR-2 phosphorylation at Y951 and subsequent recruitment of VEGF-receptor-associated proteins (VRAPs; also known as T-cell-specific adapter molecule, TSAd) (37). VRAP contains an SH2 domain and a C-terminal proline-rich motif. VRAP is constitutively associated with SRC and phosphatidylinositol 3-kinase (PI3K) (Figure 2). SRC is a non-receptor tyrosine kinase regulating cytoskeleton reorganization, metastasis, and proliferation (38). VRAP recruits and activates PI3K either directly or through SRC. PI3K then phosphorylates membrane-bound phosphatidylinositol-3,4-bisphosphate (PIP2) to phosphatidylinositol-3,4,5-triphosphate (PIP3). The binding of PIP3 to the pleckstrin homology domain of Akt leads to Akt activation. Akt has numerous and diverse biological effects by phosphorylating a variety of substrates. These effects include involvement in metabolism, protein synthesis, apoptosis pathways, transcription factor regulation, and cell cycle regulation (39–41). The overall effect of Akt activation is antiapoptosis or cell survival. In conclusion, VEGFR-2 cross talks with SRC or PI3K/Akt are mediated by VRAP/TSAd, and these cross talks are important to VEGF-induced cytoskeletal reorganization, migration, cell survival, and proliferation (34).

### Phospholipase C-γ

In porcine aortic endothelial cell models and human umbilical vein endothelial cell models, VEGFR-2 pY1175 recruits and activates phospholipase C-γ (PLC-γ), which is essential for generating inositol phosphates (35, 42). PLC-γ hydrolyzes PIP2 to release second messengers 1,2-diacylglycerol (DAG) and inositol 1,4,5-trisphosphate (IP3). DAG is an activator of protein kinase C (PKC). PKC activates extracellular signal regulated kinases 1/2 (ERK1/2), which result in cell survival (Figure 2). IP3 binds to its receptor IP3R in endoplasmic reticulum to release Ca^2+^ from endoplasmic reticulum to cytoplasm. Ca^2+^ activates calmodulin, which further activates calcineurin. Calcineurin facilitates calcium-sensitive nuclear factor of activated T-cells (NFAT) to promote cell proliferation. Aforementioned VEGFR-2 adaptor VRAP can also activate PLC-γ. Moreover, the essential *in vivo* role of PLC-γ in vasculogenesis has been verified in a mutant Vegfr-2 Y1173F knock-in mouse model (murine Y1173 corresponding to Y1175 in human VEGFR-2). Vegfr-2 Y1173F mice died between embryonic days 8.5 and 9.5 without any organized blood vessels or yolk sac blood islands, and hematopoietic progenitors were severely reduced, phenotypically mimicking Vegf2^−/−^ mice (43).

### SH2 Domain-Containing Adaptor Protein B

In pig aortic endothelial cells expressing human VEGFR-2 molecules, VEGF stimulation induces VEGFR-2 phosphorylation at Y1175, and pY1175 recruits SH2 domain-containing adaptor protein B (SHB) (44). SHB activates focal adhesion kinase (FAK), which is a highly conserved tyrosine kinase regulating focal adhesions (Figure 2). FAK activates small Rho GTPase RAC1, which drives actin polymerization, forms lamellipodia, and promotes cell migration (45, 46). Furthermore, *Shb*^−/−^ mouse model has revealed the essential *in vivo* role of SHB in vasculogenesis. *Shb*^−/−^ mice have abnormal endothelial ultrastructures in liver sinusoids and heart capillaries (47).

### Neuronal CDK

In porcine aortic endothelial cell models and human umbilical vein endothelial models, VEGFR-2 pY1214 recruits SH2/SH3 adaptor protein neuronal CDK (NCK) (48, 49). NCK activates SRC family kinase FYN (36). FYN activates p21-activated protein kinase-2 (PAK-2), and PAK-2 activates CDC42 (36). CDC42 subsequently activates p38 mitogen-activated protein kinase (MAPK) (50). p38 MAPK is a stress-activated protein kinase, and its activation is known to promote VEGF-triggered stress fiber formation and endothelial cell migration in human umbilical vein endothelial cells (51). To conclude, VEGFR2 recruits NCK/FYN to activate p38, which promotes stress fiber formation and cell migration (Figure 2).

The knowledge of VEGFR-2 signaling gained from endothelial cell model may be applied to VEGFR-2 signaling in cancer cells. For example, PLC-γ activating mutant R707Q is observed in human primary cardiac angiosarcoma. PLC-γ R707Q leads to a hyperactive VEGFR-2 signaling and increases apoptotic resistance in cancer cells (52). Furthermore, *Shb*^−/−^ mice have impaired tumor growth (47).

## VEGFR-2 Signaling in Breast Cancer Cells

### The Regulation of VEGFR-2 Expression in Breast Cancer

**Figure 3:**
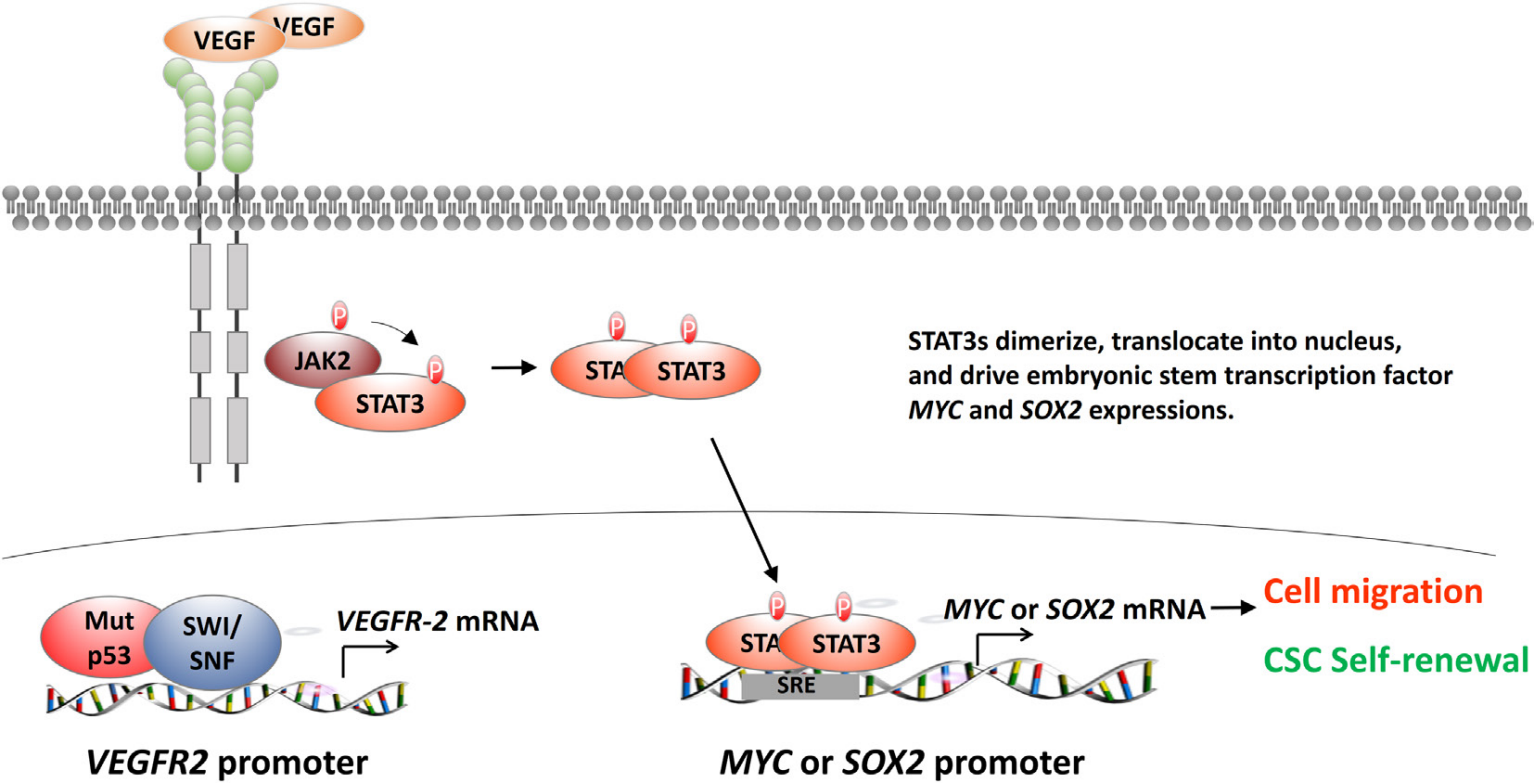
**The regulation of VEGFR-2 in breast cancer cells**. After the binding of VEGF to its cognate receptor VEGFR-2, VEGFR-2 activates STAT3 dimer formation. STAT3 dimer activates STAT3-response element (SRE)-containing genes, including *MYC* and *SOX2* (top and lower right). MYC and SOX2 are embryonic stem cell transcription factors (ES-TF). They will affect many downstream EMT-related genes, such as up-regulating *SNAIL*, *SLUG*, *ZEB1*, and *ZEB2* and down-regulating *CDH1*. These genes will endow the breast cancer cells the capability of cell motility and cancer stem cell self-renewal. Another exciting advance found that mutant p53, together with SWI/SNF histone remodeling complex, will be recruited to the *VEGFR-2* promoter to activate VEGFR-2 in triple-negative breast cancer cell lines (lower left).

Vascular endothelial growth factor receptor-2’s regulatory role for cancer development is largely unknown. Pfister et al. recently identified that mutant p53 activates *VEGFR-2* gene expression (53). Mutant p53 and histone remodeling complex switch/sucrose non-fermentable (SWI/SNF) colocalize to the *VEGFR-2* promoter. SWI/SNF remodels the *VEGFR-2* promoter and keeps the promoter at an “open” configuration (Figure 3, bottom left). Pfister et al. also revealed an interesting aspect of the relationship between SWI/SNF and mutant p53 from RNA-seq analyses. The results showed that more than 40% of mutant p53-regulated genes are also under the regulation of SWI/SNF (53). It is inconclusive whether SWI/SNF coactivates all types of mutant p53s due to limited cell lines and mutation types assayed in this study. This study is important because it identifies SWI/SNF as a general cofactor of p53 mutants. Disrupting the mutant p53-SWI/SNF interaction would be an effective strategy in treating triple-negative breast cancer.

More importantly, Pfister et al. showed that VEGFR-2’s expression is critical for mutant p53-containing breast cancer cell growth and migration (53). The results should be appreciated because Pfister et al. extensively used mammosphere assay and other three-dimensional (3D) culture techniques (53). These 3D techniques are commonly accepted as a good indicator for clinical response. These *in vitro* data support that targeting VEGFR-2 might be beneficial as a cancer stem cell therapy.

### The Role of VEGFR-2 Signaling in Breast Cancer Stem Cells

Interestingly, Zhao et al. explored the role of VEGFR-2 in CSCs using established triple-negative breast cancer cell lines, dissociated primary breast tumor cells, and mice xenograft models (19). Zhao et al. found that VEGF-triggered VEGFR-2 activation increases mammospheres and aldehyde dehydrogenase activity in triple-negative breast cancer lines and dissociated primary cancers *in vitro*. EMT generates cancer cells with stem cell properties (54). Next, Zhao et al. focused on the role of VEGFR-2 in CSC populations and found that VEGFR-2 increases breast cancer CSCs, orthotopic tumors, and metastasis *in vivo* (19). More importantly, Zhao et al. delineated the downstream signaling of VEGFR-2 in CSCs (19). Zhao et al. found that VEGFR-2 recruits Janus-family tyrosine kinase 2/signal transducers and activators of transcription 3 (JAK2/STAT3) and STAT3 dimer induces *MYC* and *SOX2* expression (Figure 3, top and bottom right). Meta-analysis of over a thousand primary breast cancers showed that high VEGF expression is strongly associated with STAT3 and MYC expression, supporting the link between VEGFR-2 and CSC self-renewal (19).

The regulation of breast cancer CSCs by VEGFR-2 is an important finding, which further supports preclinical investigation of anti-VEGFR-2 in breast cancer treatments. Targeting CSC-addicted signaling pathways is attractive. Increasing evidence suggests that cells within a tumor can exhibit heterogeneity and cancer originates from CSCs (55, 56). CSCs are thought to be responsible for many attributes of cancer, including radiation resistance/chemoresistance, metastasis, and relapse of disease (57, 58). Targeting VEGFR-2 and/or downstream JAK2 or STAT3 might overcome the radiation resistance and chemoresistance in triple-negative breast cancer by eliminating CSCs.

## Implications of Targeting VEGFR-2 for Treating Breast Cancer

Breast cancer is the most frequent cancer and the second-most common cause of death from cancer in women worldwide (59). Two thirds of new breast cancers express estrogen receptor α (ER) protein, and the growth of these primary tumors is predominantly depend on estrogen (60). Till date, Food and Drug Administration (FDA) has proved three selective ER modulators (SERMs) – raloxifene, toremifene, and tamoxifen – and three aromatase inhibitors (AIs) – anastrozole, letrozole, and exemestane (61). Although patients with ER-positive breast cancers can be treated with these drugs successfully, it is in the treatment of triple-negative (ER^−^, PR^−^, Her2^−^) breast cancer where there is a clear demand for the development of new therapies (62). Triple-negative breast cancers commonly cause mortality when these tumors metastasize to distant organs including lung and brain. Bone metastasis can cause significant morbidity.

Anti-VEGF therapy in metastatic breast cancer was initially embraced with great enthusiasm. Two commonly used reagents are bevacizumab (Avastin) (27), an anti-VEGF monoclonal antibody, and its antibody derivative ranibizumab (Lucentis) (63). However, anti-VEGF therapy for breast cancer has been a “veritable roller coaster of results” (64). Bevacizumab, initially on FDA “fast track” for metastatic breast cancer, was revoked of approval in breast cancer in 2011 (65). The efficacy of bevacizumab in breast cancer is unclear. Bevacizumab delayed metastatic breast progression in early trials with paclitaxel, whereas subsequent trials showed no increase in overall survival (66). Moreover, other preclinical studies suggested that bevacizumab promotes more aggressive metastatic behavior in surviving cells (67, 68). Causes of resistance to bevacizumab are that bevacizumab reduces tumor vessel supply, decreases drug penetration, and increases hypoxia to stimulate even greater VEGF production to overcome drug effects (69).

To overcome drug resistance to bevacizumab, chemical inhibitor drugs against VEGFR-2 may be proven effective. Indeed, FDA has proved several small compound drugs inhibiting VEGFR-2, including sunitinib (Sutent) (70–72), sorafenib (Nexavar) (73–75), axitinib (Inlyta) (76), and pazopanib (Votrient) (77, 78). The development of these VEGFR-2 inhibitors supports the further investigation of their clinical benefits for a selective subset of breast cancer patients whose mutant p53 activates VEGFR-2. At present, the benefit of sunitinib in breast cancer is undefined. Sunitinib had shown single-agent activity in the treatment of metastatic breast cancer (79). In other trials, sunitinib has failed to demonstrate therapeutic benefit in either first-line or refractory breast cancer (80, 81). The clinical experience with sorafenib in breast cancer is limited, with only a recently completed phase I/II trial of combination of sorafenib and anastrozole (NCT00217399). Axitinib has significant benefits only in patients who have previously received paclitaxel (NCT00076024), which suggested anti-VEGFR-2 therapy might best work on patients receiving prior paclitaxel (82). Similar to axitinib, pazopanib showed additional benefits to paclitaxel treatment in breast cancer from a recent clinical trial (NCT01644825). Pazopanib plus paclitaxel group has a significant longer progression-free survival than the paclitaxel only group [median, 6.35 months (95% CI, 5.36–11.02) versus 3.49 months (2.01–5.66); hazard ratio, 0.42 (95% CI, 0.25–0.69); *p* = 0.0002] (83). To reduce the complexity of drug response, it is of particular interest to identify the subgroups of breast cancer patients who will preferentially benefit from anti-VEGFR-2 therapy with combinations of biomarkers. Mutant p53 could be one of the predictive biomarkers.

## Conclusion

Much progress has been made in understanding the biology of VEGFR-2 in breast cancer. One breakthrough is that mutant p53 recruits SWI/SNF to activate *VEGFR-2* expression (53). This finding suggests compounds disrupting mutant p53–SWI/SNF interaction might be effective in treating breast cancer. Another interesting study found that VEGFR-2 recruits JAK2/STAT3 to activate embryonic stem cell transcription factors *MYC* and *SOX2* in breast cancer CSCs (19). This finding offers another possibility that VEGFR-2 inhibitor and/or JAK2/STAT3 inhibitors could be used as a cancer stem cell-targeted therapy in breast cancer.

## Conflict of Interest Statement

The authors declare that the research was conducted in the absence of any commercial or financial relationships that could be construed as a potential conflict of interest.
